# Both Biosynthesis and Transport Are Involved in Glucosinolate Accumulation During Root-Herbivory in *Brassica rapa*

**DOI:** 10.3389/fpls.2019.01653

**Published:** 2020-01-10

**Authors:** Axel J. Touw, Arletys Verdecia Mogena, Anne Maedicke, Rebekka Sontowski, Nicole M. van Dam, Tomonori Tsunoda

**Affiliations:** ^1^ Molecular Interaction Ecology, German Center for Integrative Biodiversity Research (iDiv) Halle-Jena-Leipzig, Leipzig, Germany; ^2^ Institute of Biodiversity, Friedrich Schiller University Jena, Jena, Germany; ^3^ Research and Development Department, Center for Genetic Engineering and Biotechnology, Camagüey, Cuba; ^4^ Faculty of Agriculture and Life Science, Shinshu University, Kamiina-County, Japan

**Keywords:** cabbage root fly, plant–insect interactions, above–belowground interactions, induced plant responses, optimal defense theory

## Abstract

The optimal defense theory predicts that plants invest most energy in those tissues that have the highest value, but are most vulnerable to attacks. In *Brassica* species, root-herbivory leads to the accumulation of glucosinolates (GSLs) in the taproot, the most valuable belowground plant organ. Accumulation of GSLs can result from local biosynthesis in response to herbivory. In addition, transport from distal tissues by specialized GSL transporter proteins can play a role as well. GSL biosynthesis and transport are both inducible, but the role these processes play in GSL accumulation during root-herbivory is not yet clear. To address this issue, we performed two time-series experiments to study the dynamics of transport and biosynthesis in local and distal tissues of *Brassica rapa*. We exposed roots of *B. rapa* to herbivory by the specialist root herbivore *Delia radicum* for 7 days. During this period, we sampled above- and belowground plant organs 12 h, 24 h, 3 days and 7 days after the start of herbivory. Next, we measured the quantity and composition of GSL profiles together with the expression of genes involved in GSL biosynthesis and transport. We found that both benzyl and indole GSLs accumulate in the taproot during root-herbivory, whereas we did not observe any changes in aliphatic GSL levels. The rise in indole GSL levels coincided with increased local expression of biosynthesis and transporter genes, which suggest that both biosynthesis and GSL transport play a role in the accumulation of GSLs during root herbivory. However, we did not observe a decrease in GSL levels in distal tissues. We therefore hypothesize that GSL transporters help to retain GSLs in the taproot during root-herbivory.

## Introduction

In their role as primary producers plants form the basis of most natural communities. Consequently, plants are involved in interactions with many different organisms, including aboveground and belowground herbivores. To limit the negative effects of herbivory, plants have evolved an elaborate defense system, including structural traits such as thorns and trichomes, antidigestive proteins, and an extensive arsenal of defense-related metabolites (Schoonhoven et al., 2005). The classes of these metabolites vary by taxon and are often characteristic for distinct plant families (reviewed in Piasecka et al., 2015).

Glucosinolates (GSLs) are a class of well-studied defense metabolites that are characteristic for brassicaceous plants. They are derived from amino acids, and are broadly divided into three groups based on their amino acid precursor (reviewed in Sønderby et al., 2010). Indole GSLs have a side chain derived from tryptophan, aliphatic GSLs from methionine and benzyl GSLs from phenylalanine or tyrosine. GSLs are stored in the vacuoles of specific cells (Kissen et al., 2009). Upon herbivore damage, GSLs mix with the enzyme myrosinase, which is stored in separate cells. This leads to the formation of breakdown products, the structure and biological activity of which strongly depend on the structure of the GSL. In general, the hydrolysis of indole GSLs leads to the formation of instable isothiocyanates (ITCs) and nitriles, whereas aliphatic and benzyl GSLs mostly produce noxious ITCs (Wittstock and Burow, 2010). Due to this difference in breakdown products, structurally different GSL groups cause resistance against distinct groups of attackers. In general, indole GSLs act against phloem feeders and pathogens (Kim et al., 2008; Bednarek et al., 2009), whereas aliphatic, indole and benzyl GSLs can affect the performance of chewing insects (Beekwilder et al., 2008; Schlaeppi et al., 2008; Bejai et al., 2012).

GSLs are constitutively present in all tissues of brassicaceous plants (Wittstock and Gershenzon, 2002), but quantitative and qualitative differences in GSL composition occur between different plant parts. Constitutive GSL concentrations are generally higher in roots compared to shoots (reviewed in van Dam et al., 2009). Moreover, GSLs are differentially distributed over different organs. Recent studies showed that the distribution of GSLs over different parts follows optimal defense theory (ODT) (Tsunoda et al., 2018). The ODT predicts that plants allocate defenses preferentially to the plant parts that are highly attractive to potential attackers and are most valuable to the plant at the same time (McKey, 1974; Meldau et al., 2012). This implies that in aboveground tissues young leaves and reproductive organs, such as flowers and seeds, contain the highest GSL concentrations. In belowground tissues, constitutive GSLs accumulate mainly in the tap- and lateral roots, whereas GSL levels are lower in fine roots (Tsunoda et al., 2017). In addition, GSLs accumulate in damaged tissue in response to insect herbivory (van Dam and Raaijmakers, 2006). The strength of this induced response and the composition of the resulting GSL profile depends in large on the feeding guild of the attacker. Feeding by chewing herbivores such as beetles, caterpillars and fly larvae generally leads to strong increases in total GSL levels (reviewed in Textor and Gershenzon, 2009). In contrast, sucking insects such as aphids do not induce GSL accumulation, or in some cases even inhibit production of certain GSL classes (Kim and Jander, 2007). Similar to the allocation of constitutive defenses, induced plant responses to herbivory follow ODT predictions (reviewed in Meldau et al., 2012). In shoot tissues of *Nicotiana sylvestris*, accumulation of nicotine is more inducible in younger leaves (Ohnmeiss and Baldwin, 2000). In belowground tissues of *Brassica*, the taproot responds more strongly to root-herbivory compared to lateral and fine roots, leading to accumulation of high GSL levels in the taproot (Tsunoda et al., 2018). In line with the ODT, herbivore damage on the taproot had a larger impact on plant biomass than herbivore feeding on fine roots (Tsunoda et al., 2018).

The distribution of GSLs over the plant is the combined result of several, tightly coordinated processes. Increased local biosynthesis plays an important part in GSL accumulation upon induction (Tytgat et al., 2013), whereas transport of GSLs from other parts towards the feeding site may play a role as well (Johnson et al., 2016). In undamaged plants, long-distance transport of GSLs to designated plant parts is regulated through the activity of GSL transporter proteins (GTRs) (Nour-Eldin et al., 2012; Andersen et al., 2013; Madsen et al., 2014). The role of GTRs is twofold: they either play a role in selective loading of GSLs into the phloem for transport to other plant compartments, or by retaining GSLs in certain parts by preventing transport *via* the xylem (Madsen et al., 2014; Jørgensen et al., 2017). Because of their partial substrate specificity, GTRs can fine-tune the distribution of GSLs belonging to different classes and of GSLs of different chain lengths (Andersen et al., 2013).

Since the production of GTRs is inducible, biotic and abiotic factors can affect the allocation of GSLs over specific plant parts (Nour-Eldin et al., 2012). However, how transport and biosynthesis act in concert to change GSL accumulation patterns in plant–herbivore interactions has not been studied so far (Jørgensen et al., 2015; Burow and Halkier, 2017). The aim of this study was to explore the temporal and spatial dynamics of GSL accumulation and the underlying molecular mechanisms during root herbivory in line with ODT. We expected that the accumulation of GSLs in response to root herbivory would not only be the result of local biosynthesis, but also of active transport from distal tissues. We tested this hypothesis in two time course experiments using wild mustard plants (*Brassica rapa*) and the cabbage root-fly (*Delia radicum*), a specialist root-herbivore on brassicaceous plants. Adult females typically oviposit on the lower part of the stem. After hatching, the larvae mine into the taproot, where they cause extensive damage. Because of this feeding behavior, GSL induction is mainly seen in the taproot (Tsunoda et al., 2018). This makes *D. radicum* an attractive organism to study local and systemic defense induction in belowground tissues. In the first experiment, we investigated the effects of root-herbivory on the accumulation of GSLs in both above- and belowground organs after 3 and 7 days, when GSL accumulation is known to occur (Tsunoda et al., 2018). In the second experiment, we focused on the dynamics of the molecular mechanisms underlying this accumulation at earlier time points after the onset of herbivory, and therefore sampled at 12 and 24 h after infestation. Specifically, we tested the following hypotheses: (i) there is a negative correlation between allocation of GSL to local- and distal tissues (ii) transporter genes are expressed earlier than biosynthesis genes and (iii) transporter genes are expressed earlier in distal root tissues than in damaged root tissues.

## Materials and Methods

### Plants and Insects

*Brassica rapa* seeds used during the first experiment were bought from a commercial supplier (Horti Tops, the Netherlands), whereas the seeds used during the second experiment were collected in 2009 from a wild population in Maarsen (the Netherlands) (Danner et al., 2015). Both *B. rapa* varieties are fast-cycling, which flower without vernalization. The seeds were germinated on fine-grained vermiculite in plastic containers. The containers were kept in a climate chamber (E-36L Reach in Plant Growth Chamber, CLF Plant Climatics GmbH, Wertingen, Germany) at 20°C (16:8 h day:night) and 60% relative humidity for one week. After germination, the seedlings were transplanted to 2.5 L pots filled with river sand and placed in a greenhouse chamber belonging to the botanical gardens of Leipzig University (Leipzig, Germany) at 27°C (day, 16 h) and 21°C (night, 8 h) at 50% relative humidity. Two batches of sand that were used during transplanting were weighed, dried for 24 h at 50°C and weighed again to determine initial water content. After transplanting, the seedlings were supplied with 2P Hoagland solution (double KH_2_PO_4_ compared to regular Hoagland solution) (Van Dam et al., 2004) so that the total water content of the sand amounted to 14% w/w of the dry mass. Every 2–3 days, five randomly chosen pots were weighed to determine the volume of water needed to keep the water content of the sand at 14%. Once a week, plants were watered with 2P Hoagland solution instead of water. Plants were placed in a full-factorial block design with time point of harvest as the blocking factor. Within each block, control plants were paired with treatment plants of similar size and habit.

*Delia radicum* L. (Diptera: Anthomyiidae) larvae used during the experiments originated from our rearing, which was established 4 years ago. The rearing was started from a culture kindly provided by Dr. Anne-Marie Cortesero (University of Rennes, France). The colony has been maintained since in a climate chamber at 20°C (16:8 h day:night) on cabbage turnip (*Brassica oleracea*). Second instar larvae were used during the experiments.

### Experimental Design

We tested the defense response of *B. rapa* to herbivory by *D. radicum* in two experiments. The experimental setup of both experiments was based on a paired block design with six biological replicates per treatment group. The two factors were root herbivory and duration of root herbivory. The root herbivory treatment had two levels: control (no larvae) or infestation by three second-instar *D. radicum* larvae. The duration of root herbivory had two levels in the first experiment (3 and 7 days) and four levels in the second experiment (12 h, 24 h, 3 days and 7 days).

Infestation with *D. radicum* took place after around 40 days after transplanting the seedlings to the pots. This coincided with the moment when plants had developed three leaf pairs (BBCH code 13, according to Feller et al., 1995). Separate sets of plants were destructively harvested 3 and 7 days after the start of herbivory in the first experiment, and after 0.5, 1, 3, and 7 days in the second. During harvest, plants were first split in above- and belowground parts by cutting the stem immediately above the taproot using garden scissors. After flushing the sand out with cold water, the root systems were split into two organs: fine roots and taproots. Fine roots were collected from the lower half of the root system to clearly separate them from lateral or taproots. The shoots were split into two organs: leaf lamina and stem or hypocotyl. We divided leaves into three groups: young leaves (two most recently developed leaves), mature leaves (two leaves directly below the young leaves) and old leaves (two leaves directly below the mature leaves). In the first experiment, one leaf of each group were pooled together for later analysis. After harvest, the separate plant organs were wrapped in aluminum foil, flash-frozen in liquid nitrogen and stored at -80°C. Afterwards, we finely ground each sample in liquid nitrogen using a mortar and pestle. For the first experiment two of the six plants belonging to the same treatment were pooled, resulting in three biological replicates per treatment (n = 3). For the second experiment all six biological replicates were analyzed individually (n = 6).

### Gene Expression Analysis

Total RNA was extracted from ±100 mg ground plant tissue following a protocol adapted from Oñate-Sánchez and Vicente-Carbajosa (2008). The extracted RNA was subsequently treated with DNAse I (Thermo Scientific, Waltham, MA, USA) following the manufacturer’s instructions. The quality of RNA was checked visually by gel-electrophoresis and by measuring 260/230 and 260/280 absorbance ratios using a NanoPhotometer^®^ P330 (Implen, Munich, Germany). Next, first-strand cDNA was synthesized from 1 μg purified total RNA using Revert Aid H minus reverse transcriptase (Thermo Scientific, Waltham, MA, USA) following the manufacturer’s instructions. The samples were incubated at 42°C for 60 min, 50°C for 15 min, and finally 70°C for 15 min in a thermal cycler (Techne, Stone, UK). Real-time quantitative PCR (RT-qPCR) procedures were performed on a CFX384 Real-time system (BioRad, Munich, Germany) using the gene-specific primers as described in **Table S1**. The qPCR conditions were: 2 min at 50°C, 5 min at 95°C, and 40 cycles of 30 s at 95°C, 30 s at 58°C, 45 s at 72°C. Three technical replicates were analyzed per gene for each of the three biological replicates in experiment 1 and of the six biological replicates in experiment 2. Relative expression of target genes was calculated using the comparative 2^-ΔΔCT^ method as described in Livak and Schmittgen (2001). The data was normalized to expression of the housekeeping gene *GAPDH* in the first experiment and *ACTIN 7* in the second experiment. Expression levels were then normalized to those in the control plants. The genes selected for this study play a role in either GSL biosynthesis or transport. *CYP83A1* (CYTOCHROME P450, FAMILY 83, SUBFAMILY A, POLYPEPTIDE 1) is involved in the biosynthesis of aliphatic GSLs. *CYP79B2* (CYTOCHROME P450, FAMILY 79, SUBFAMILY B, POLYPEPTIDE 2) is involved in the biosynthesis of indole GSLs. *GTR1A2* and *GTR2A2* (GLUCOSINOLATE TRANSPORTER 1&2) regulate transport of aliphatic and indole GSLs, whereas *GTR3A1* exclusively regulates transport of indole GSLs (Jørgensen et al., 2017). Primer sequences used in this experiment are shown in **Table S1**.

### Glucosinolate Analysis

GSL extraction was performed following the method as described in Grosser and van Dam (2017). In brief, freshly ground plant tissue was freeze-dried, after which ±100 mg of material was used for extraction from three biological replicates in experiment 1 and from six biological replicates in experiment 2. GSLs were extracted in 70% methanol at 90°C, after which the supernatant was transferred to an ion-exchange column with Sephadex G-25 (Merck, Darmstadt, Germany) as column material. After washing the extracts with 70% methanol and adding a NaOAc buffer to the column, sulfatase (from Helix pomatia type H-1, Merck, Darmstadt, Germany) was pipetted onto the extracts to remove the sulfate group from the GSLs. The desulfo-GSLs that were released from the ion-exchange column as a result of sulfatase activity were eluted in ultrapure water and collected. Next, the samples were freeze-dried and re-dissolved in 1 ml of ultrapure water. The GSLs in the samples were separated using a reversed phase high-pressure liquid chromatography (HPLC) set-up equipped with a photodiode array detector (PDA; Thermo Scientific Ultimate 3000 series) at wavelengths of 229 nm and 272 nm. A reversed-phase Acclaim™ 300 C18 column (4.6 × 150 mm, 3 μm, 300 Å, Acclaim 300, Thermo Fisher Scientific) was used for separation with 100% H_2_O (solvent A) and 99% acetonitrile in water (solvent B) as solvents. The separation conditions were as follows: equilibration took place at a gradient profile of 98% of solvent A for 4.3 min, followed by a gradient to 35% solvent B within 24.3 min, and a hold until 29°min at 35% solvent B. Next, the gradient went back to the initial 98% of solvent A within 1 min and held at initial conditions for 10 min at a flow of 0.6 ml/min. Desulfo-GSLs were identified based on retention time and UV spectra compared to commercially available reference standards (Phytoplan, Heidelberg, Germany). We used sinigrin as an external standard for GSL quantification. The resulting data were processed using Chromeleon 7.2 SR5 MUa (9624; Thermo Fisher Scientific, Waltham, MA, USA). Response factors and approximate retention times of each GSL are shown in **Table S2**. Detailed results of individual GSL accumulation are shown in **Table S3** for the first experiment and **Table S4** for the second experiment.

### Statistical Analysis

All statistical analyses were performed with version 3.4.3 of R (RStudio Team, 2018). Normality of the data and homogeneity of variance were inspected visually using QQ- and residual plots. When the assumptions were not met, the respective data were log-transformed. Within each plant organ, concentrations of total GSLs and of each GSL class individually, and of transcript accumulation of each gene were analyzed by two-way ANOVA with treatment and time as fixed factors. When either factor had a significant effect, student’s t-tests where used to test for significant differences between treatments at individual time points. Samples that could not be analyzed due to technical problems during sample processing were treated as missing values.

## Results

### Experiment 1: Late Time-Points

#### Indole GSLs Accumulate in the Taproot During Root Herbivory

**Figure 1:**
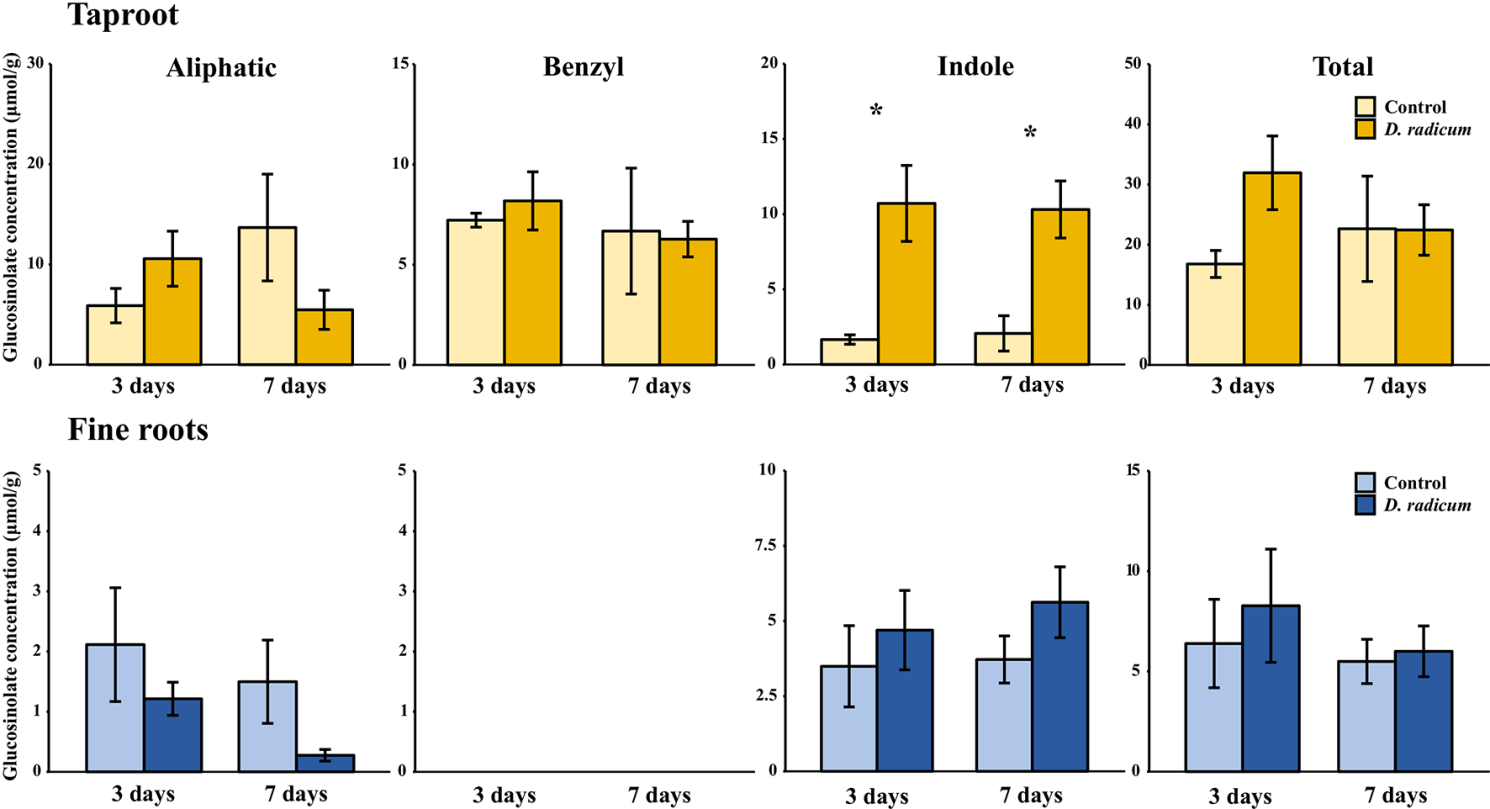
Concentration (μmol/g dry mass) of aliphatic, benzyl, indole and total GSLs in the tap root and fine roots of *B. rapa* plants after 3 and 7 days of root-herbivory by *D. radicum* (mean (SE), *n* = 3). At each time‐point, the asterisk indicates significant differences according to student’s t-tests (P < 0.05, **Table S6**). *, P < 0.05.

In the first experiment, we studied the accumulation of GSLs in the taproot, fine roots, stem and leaf lamina after 3 and 7 days of herbivory. We did not observe any changes in the total amount of GSLs in the taproot during root-herbivory by *D. radicum*. Indole GSL levels were significantly elevated both after 3 and 7 days (**Figure 1**, P < 0.001, F = 26.10, two-way ANOVA, **Table 1**), whereas aliphatic and benzyl GSLs in the taproot were not affected by root herbivory. We did not observe any changes in total GSL levels or in the accumulation of individual GSL classes in the fine roots. Moreover, we did not detect benzyl GSLs in the fine roots, neither in control plants nor after root herbivory (**Figure 1**). In the stem, benzyl GSL levels decreased after 3 days of herbivory (**Figure S1**, P < 0.01, F = 17.45, **Table S5**) but returned to control levels after 7 days. We did not observe any changes in GSL levels in the leaf lamina (**Figure S1**).

**Table 1:** Statistical comparison of aliphatic, benzyl, indole and total GSL concentrations [(μmol/g) in the taproot and fine roots of *B. rapa* plants after 3 and 7 days of root-herbivory by *D. radicum* (two-way ANOVA, *n* = 3)]. Bold indicates significant difference P-values (P < 0.05).

GSL	Factors	Taproot					Fine roots				
		Df	Sum Sq	Mean Sq	F	P value	Df	Sum Sq	Mean Sq	F	P value
**Aliphatic**	Treatment	1	9.32	9.32	0.29	0.60	1	3.39	3.39	3.09	0.12
	Timepoint	1	5.45	5.45	0.17	0.69	1	1.82	1.82	1.66	0.23
	Treatment * Timepoint	1	124.66	124.66	3.90	0.08	1	0.08	0.08	0.07	0.80
	Residuals	8	256.04	32.01			8	8.75	1.09		
**Benzyl**	Treatment	1	0.24	0.24	0.02	0.88	–	–	–	–	–
	Timepoint	1	4.51	4.51	0.47	0.51	–	–	–	–	–
	Treatment * Timepoint	1	1.40	1.40	0.15	0.71	–	–	–	–	–
	Residuals	8	77.26	9.66			–	–	–	–	–
**Indole**	Treatment	1	224.40	224.40	26.10	**9.20E-04**	1	7.24	7.24	1.73	0.22
	Timepoint	1	0.00	0.00	0.00	1.00	1	1.00	1.00	0.24	0.64
	Treatment * Timepoint	1	0.50	0.50	0.06	0.82	1	0.37	0.37	0.09	0.77
	Residuals	8	68.79	8.60			8	33.39	4.17		
**Total**	Treatment	1	151.50	151.51	1.41	0.27	1	1.61	1.61	0.36	0.57
	Timepoint	1	5.30	5.32	0.05	0.83	1	20.97	20.97	4.65	0.06
	Treatment * Timepoint	1	199.00	198.96	1.85	0.21	1	0.16	0.16	0.04	0.86
	Residuals	8	862.00	107.75			8	36.06	4.51		

#### Root Herbivory Affects Expression of Biosynthesis and Transporter Genes in the Taproot

**Figure 2:**
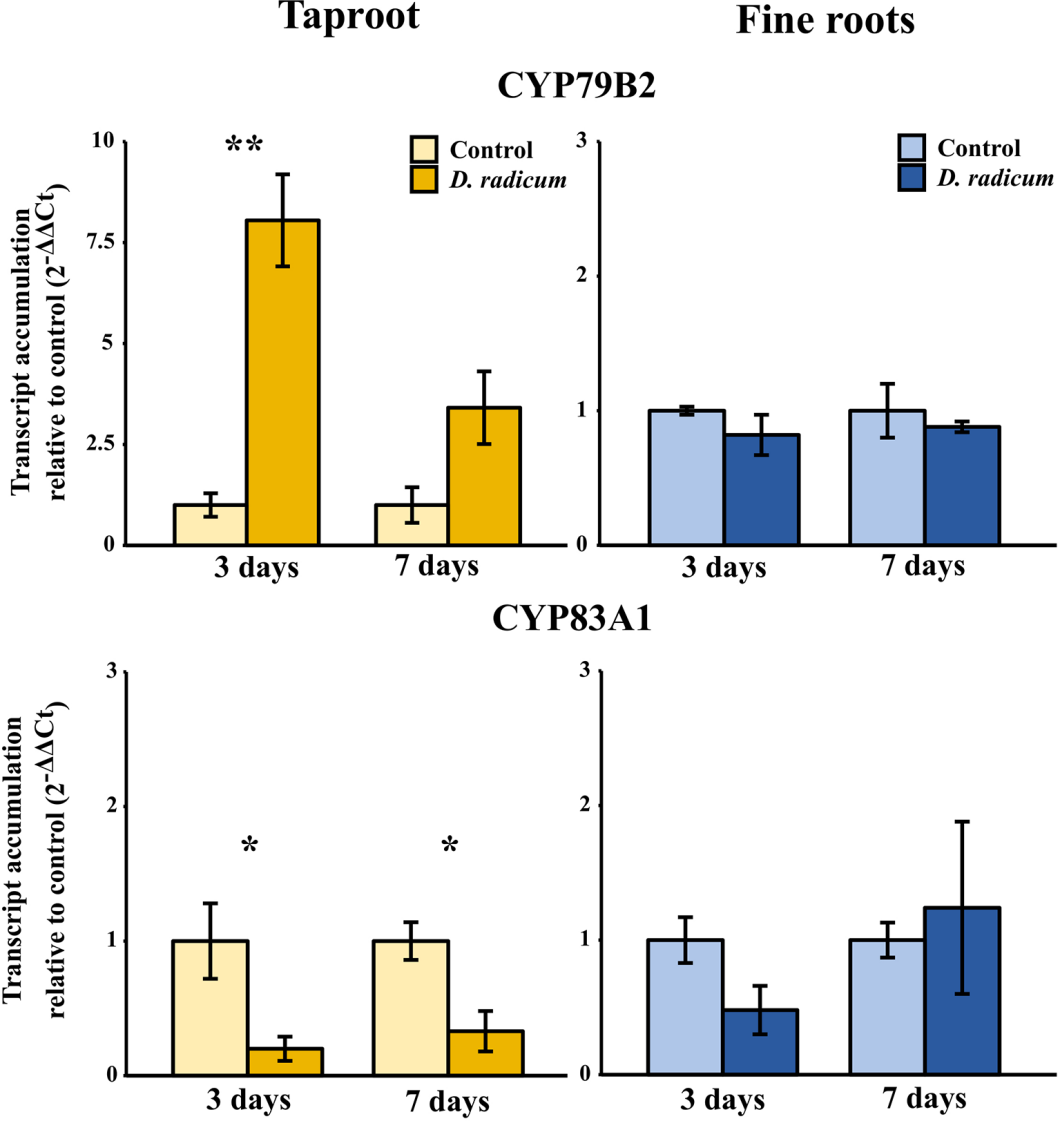
Transcript accumulation (2^-ΔΔCT^) of GSL biosynthesis genes *CYP79B2* and *CYP83A1* in the tap root and fine roots of *B. rapa* plants after 3 and 7 days of root-herbivory by *D. radicum* normalized to the housekeeping gene *GAPDH* (mean (SE), *n* = 3). At each time‐point, the asterisk indicates significant differences according to student’s t-tests (P < 0.05, **Table S8**). *, P < 0.05; **, P < 0.01.

**Figure 3:**
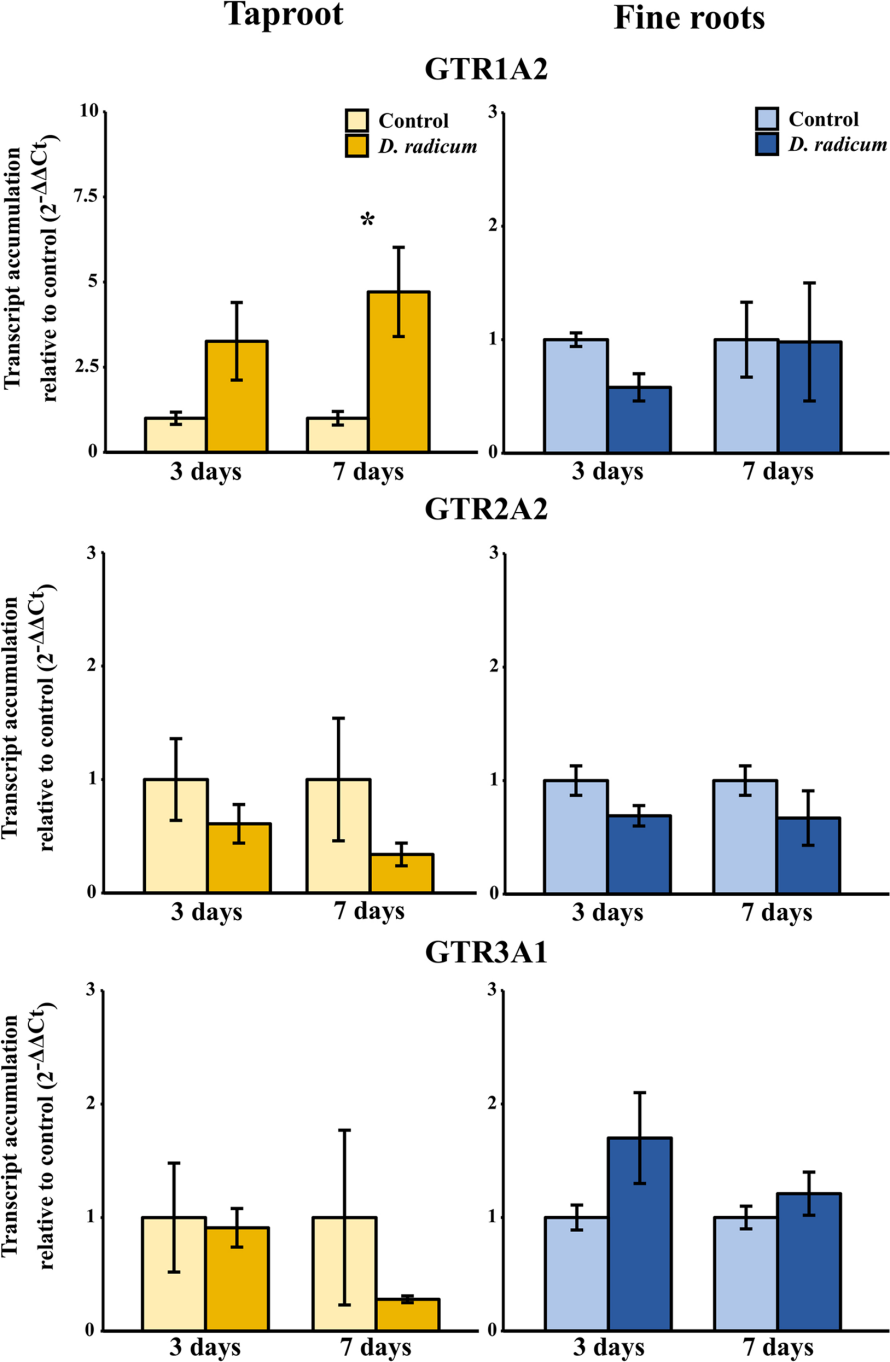
Transcript accumulation (2^-ΔΔCT^) of GSL transporters GTR 1, 2 and 3 in the tap root and fine roots of *B. rapa* plants after 3 and 7 days of root-herbivory by *D. radicum* normalized to the housekeeping gene *GAPDH* (mean (SE), *n* = 3). At each time‐point, the asterisk indicates significant differences according to student’s t-tests (P < 0.05, **Table S8**). *, P < 0.05; **, P < 0.01, ***, P < 0.001.

The observed increase in indole GSLs in the taproot coincided with an increased expression of the indole GSL biosynthesis gene *CYP79B2* (**Figure 2**, P < 0.001, F = 37.48, two-way ANOVA, **Table 2**) and downregulation of *CYP83A1* (**Figure 2**, P < 0.01, F = 16.624), which is involved in aliphatic GSL biosynthesis. In addition, we observed an increased expression of *GTR1* in the taproot after 7 days (**Figure 3**, P < 0.01, F = 11.535). No changes in the expression of biosynthesis (**Figure S2**) or transporter genes (**Figure S3**) were found in distal tissues, although a trend towards decreased expression of *GTR1* was observed in the stem after 3 days of herbivory (**Figure S3**, P = 0.08, F = 3.848, **Table S7**).

**Table 2:** Statistical comparison of transcript accumulation of GSL transporters 1, 2,and 3 and biosynthesis genes *CYP79B2* and *CYP83A1* in the taproot and fine roots of *B. rapa* plants after 3 and 7 days of root-herbivory by *D. radicum* (two-way ANOVA, *n* = 3). Bold indicates significant difference P-values (P < 0.05).

Gene	Factor	Tap root					Fine roots				
		Df	Sum Sq	Mean Sq	F	P value	Df	Sum Sq	Mean Sq	F	P value
**GTR1A2**	Treatment	1	26.74	26.74	11.535	**0.009**	1	0.145	0.145	0.485	0.506
	Timepoint	1	1.583	1.583	0.683	0.433	1	0.125	0.125	0.418	0.536
	Treatment * Timepoint	1	1.583	1.583	0.683	0.433	1	0.125	0.125	0.418	0.536
	Residuals	8	18.545	2.318			8	2.382	0.298		
**GTR2A2**	Treatment	1	0.832	0.832	2.397	0.160	1	0.307	0.307	4.089	0.078
	Timepoint	1	0.055	0.055	0.158	0.701	1	0.000	0.000	0.002	0.970
	Treatment * Timepoint	1	0.055	0.055	0.158	0.701	1	0.000	0.000	0.002	0.970
	Residuals	8	2.778	0.347			8	0.601	0.075		
**GTR3A1**	Treatment	1	0.495	0.495	0.766	0.407	1	0.629	0.629	3.793	0.087
	Timepoint	1	0.298	0.298	0.462	0.516	1	0.179	0.179	1.08	0.329
	Treatment * Timepoint	1	0.298	0.298	0.462	0.516	1	0.179	0.179	1.08	0.329
	Residuals	8	5.165	0.646			8	1.327	0.166		
**CYP79B2**	Treatment	1	67.150	67.150	37.48	**2.83E-04**	1	0.070	0.070	1.472	0.260
	Timepoint	1	16.130	16.130	9	**0.017**	1	0.003	0.003	0.056	0.820
	Treatment * Timepoint	1	16.130	16.130	9	**0.017**	1	0.003	0.003	0.056	0.820
	Residuals	8	14.330	1.790			8	0.379	0.047		
**CYP83A1**	Treatment	1	1.621	1.621	16.624	**0.004**	1	0.058	0.058	0.161	0.699
	Timepoint	1	0.013	0.013	0.134	0.724	1	0.437	0.437	1.211	0.303
	Treatment * Timepoint	1	0.013	0.013	0.134	0.724	1	0.437	0.437	1.211	0.303
	Residuals	8	0.780	0.098			8	2.885	0.361		

### Experiment 2: Early Time-Points

The results of the first experiment showed that biosynthesis and transporter genes were already significantly upregulated in the taproot by 3 days of herbivory. We therefore performed a second experiment, in which we focused on the accumulation of GSLs in the early stages of herbivory. We focused only on the taproot and fine roots since we observed hardly any changes in biosynthesis or transport dynamics in aboveground tissues.

#### Root Herbivory Affects Glucosinolates Accumulation in Local But Not in Distal Tissues

**Figure 4:**
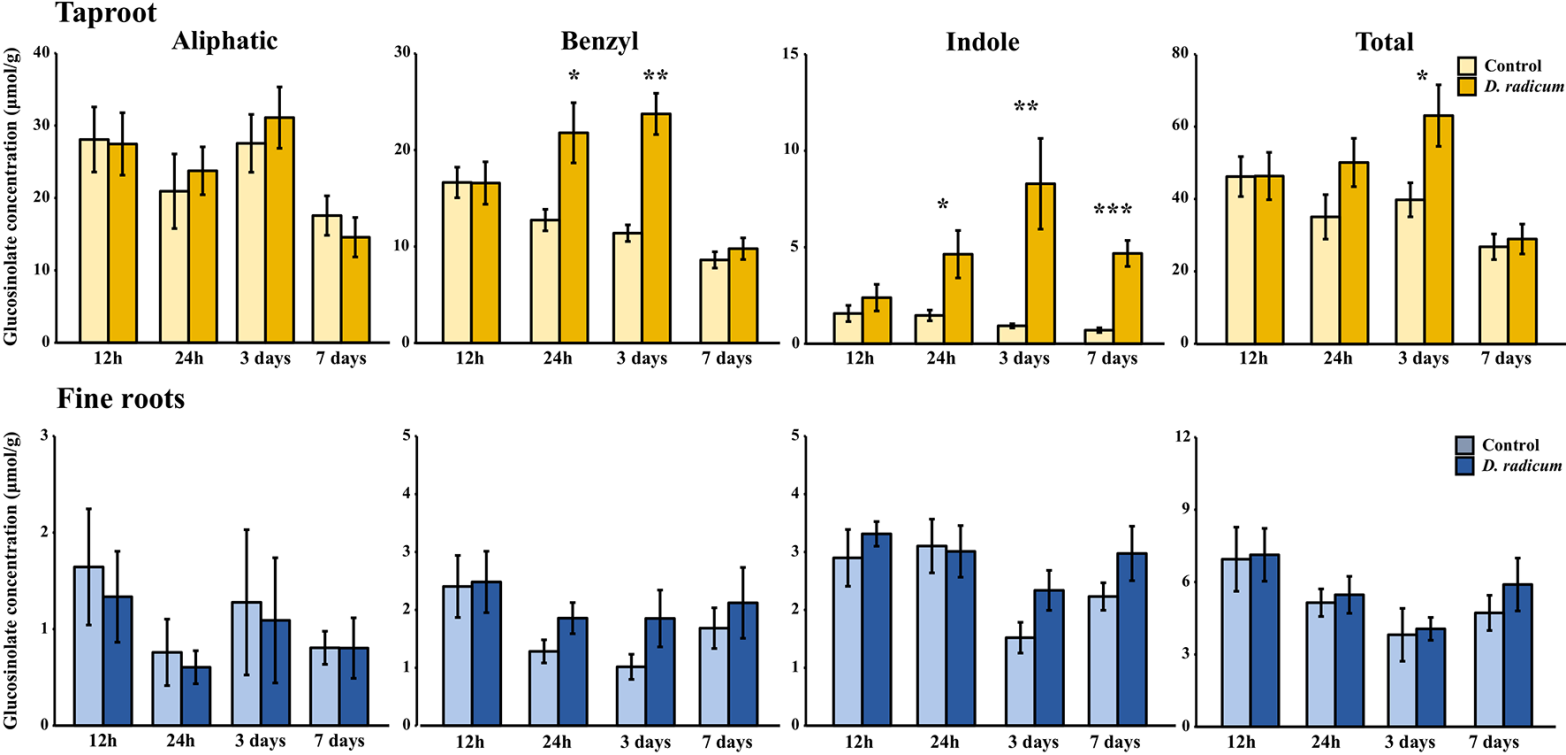
Concentration (μmol/g dry mass) of aliphatic, benzyl, indole and total GSLs in the tap root and fine roots of *B. rapa* plants after 12 h, 24 h, 3 days and 7 days of root-herbivory by *D. radicum* (mean (SE), *n* = 6). At each time‐point, the asterisk indicates significant differences according to student’s t-tests (P < 0.05, **Table S9**). *, P < 0.05; **, P < 0.01, ***, P < 0.001.

Root herbivory by *D. radicum* leads to an increased total GSL accumulation in the taproot after 3 days (**Figure 4**, P < 0.05, F = 5.80, two-way ANOVA, **Table 3**). The levels of indole GSLs (**Figure 4**, P < 0.0001, F = 30.59) and benzyl GSLs increased after 24 h and 3 days (**Figure 4**, P < 0.0001, F = 19.70), whereas aliphatic GSL levels were not affected. We did not observe any changes in GSL levels in the fine roots.

**Table 3:** Statistical comparison of aliphatic, benzyl, indole and total GSL concentrations [(μmol/g) in the taproot and fine roots of *B. rapa* plants after 12 h, 24 h, 3 and 7 days of root-herbivory by *D. radicum* (two-way ANOVA, *n* = 6)]. Bold indicates significant difference P-values (P < 0.05).

GSL	Factors	Taproot					Fine roots				
		Df	Sum Sq	Mean Sq	F	P value	Df	Sum Sq	Mean Sq	F	P value
**Aliphatic**	Treatment	1	4.00	3.70	0.04	0.84	1	0.31	0.31	0.23	0.63
	Timepoint	3	1415.00	471.50	5.04	**4.50E-03**	3	4.97	1.66	1.24	0.31
	Treatment * Timepoint	3	91.00	30.20	0.32	0.81	3	0.16	0.05	0.04	0.99
	Residuals	42	3929.00	93.50			42	56.20	1.34		
**Benzyl**	Treatment	1	371.10	371.10	19.70	**6.44E-05**	1	2.87	2.87	2.57	0.12
	Timepoint	3	643.00	214.30	11.38	**1.36E-05**	3	7.29	2.43	2.18	0.11
	Treatment * Timepoint	3	337.40	112.50	5.97	**1.74E-03**	3	0.89	0.30	0.27	0.85
	Residuals	42	791.00	18.80			42	46.95	1.12		
**Indole**	Treatment	1	184.17	184.17	30.59	**1.87E-06**	1	2.88	2.89	3.31	0.08
	Timepoint	3	44.79	14.93	2.48	0.07	3	10.73	3.58	4.10	**0.01**
	Treatment * Timepoint	3	66.04	22.01	3.66	**0.02**	3	1.58	0.53	0.61	0.62
	Residuals	42	252.86	6.02			42	36.63	0.87		
**Total**	Treatment	1	1207.00	1207.40	5.80	**2.05E-02**	1	8.10	8.10	1.29	0.26
	Timepoint	3	4068.00	1355.90	6.51	**1.02E-03**	3	40.24	13.41	2.13	0.11
	Treatment * Timepoint	3	1111.00	370.40	1.78	0.17	3	3.65	1.22	0.19	0.90
	Residuals	42	8743.00	208.20			42	264.72	6.30		

#### Root Herbivory Only Elicits Local Transcript Accumulation

**Figure 5:**
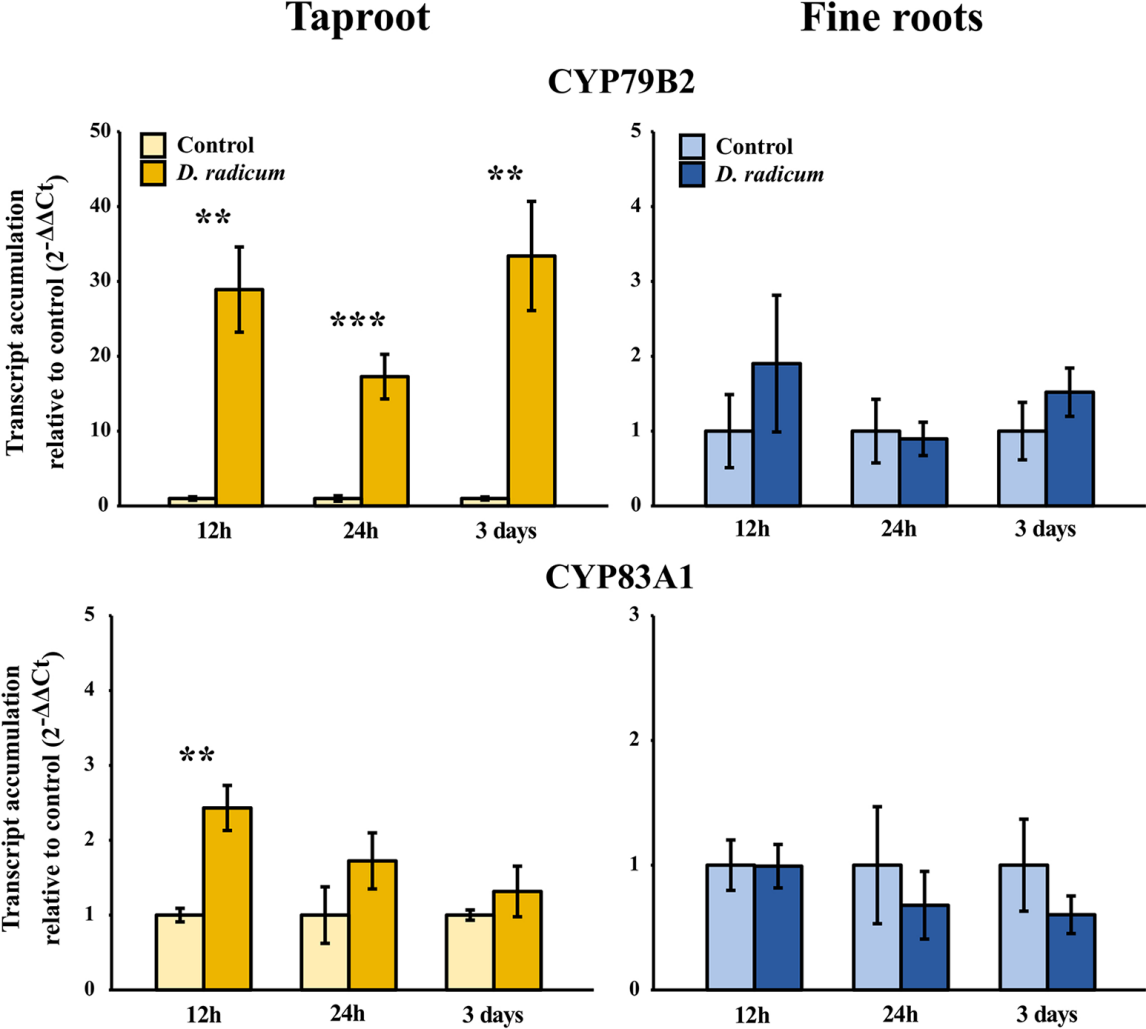
Transcript accumulation (2^-ΔΔCT^) of GSL biosynthesis genes *CYP79B2* and *CYP83A1* in the tap root and fine roots of *B. rapa* plants after 12 h, 24 h and 3 days of root-herbivory by *D. radicum* normalized to the housekeeping gene *ACTIN 7* (mean (SE), *n* = 6). At each time‐point, the asterisk indicates significant differences according to student’s t-tests (P < 0.05, **Table S10**). **, P < 0.01, ***, P < 0.001.

**Figure 6:**
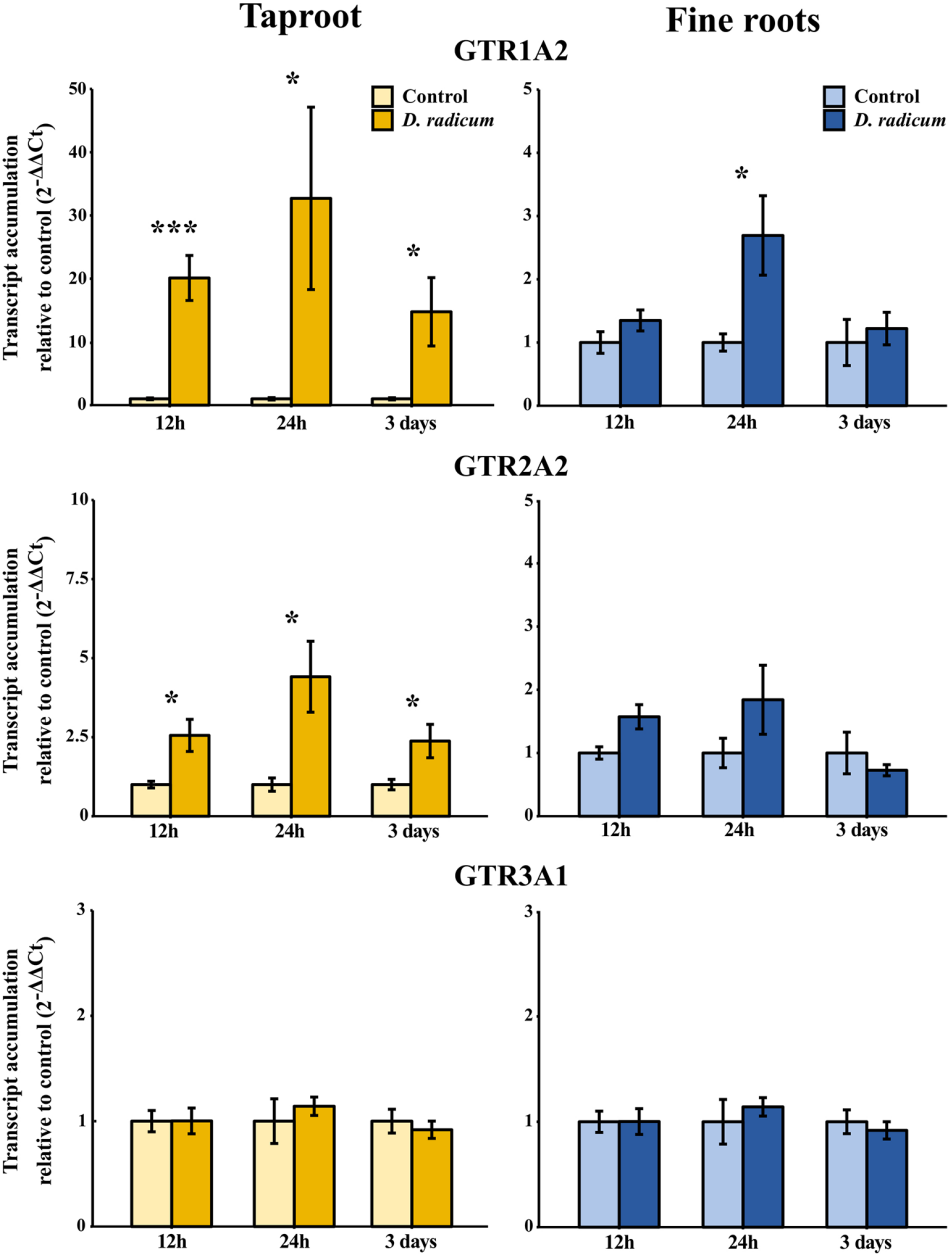
Transcript accumulation (2^-ΔΔCT^) of GSL transporters GTR 1, 2 and 3 in the tap root and fine roots of *B. rapa* plants after 12h, 24 h and 3 days of root-herbivory by *D. radicum* normalized to the housekeeping gene *ACTIN 7* (mean (SE), *n* = 6). At each time‐point, the asterisk indicates significant differences according to student’s t-tests (P < 0.05, **Table S10**). *, P < 0.05; ***, P < 0.001.

We observed a strongly increased expression of *CYP79B2* in the taproot over the entire period of herbivory (**Figure 5**, P < 0.0001, F = 61.96, two-way ANOVA, **Table 4**). In contrast, *CYP83A1* was upregulated only at 12 h after the start of root herbivory (**Figure 5**, P < 0.01, F = 12.18) and returned to control levels after 24 h. For genes involved in GSL transport, *GTR1A2* expression increased after 12 h (**Figure 6**, P < 0.001, F = 16.68) and stayed elevated until 24 h and 3 days after herbivory. The expression levels of *GTR2A2* were increased over the entire period of herbivory (**Figure 6**, P < 0.0001, F = 21.43), whereas we did not observe any changes in expression of *GTR3A1.* We did not observe any changes in expression of the biosynthesis genes *CYP83A* (**Figure 5**) or *CYP79B2* in the fine roots. The expression of the transporter *GTR1A2* increased after 24 h (**Figure 6**, P < 0.01, F = 7.68) and returned to control levels after 3 days. Root herbivory did not affect the expression of *GTR2A2* or *GTR3A1* in the fine roots (**Figure 6**).

**Table 4:** Statistical comparison of transcript accumulation of GSL transporters 1, 2, and 3 and biosynthesis genes *CYP79B2* and *CYP83A1* in the taproot and fine roots of *B. rapa* plants after 12 h, 24 h, 3 and 7 days of root-herbivory by *D. radicum* (two-way ANOVA *n* = 6). Bold indicates significant difference P-values (P < 0.05).

Gene	Factors	Taproot					Fine roots				
		Df	Sum Sq	Mean Sq	F	P value	Df	Sum Sq	Mean Sq	F	P value
**GTR1A2**	Treatment	1	4171.00	4171.00	16.68	**3.03E-04**	1	4.27	4.27	7.68	**0.01**
	Timepoint	2	509.00	254.00	1.02	0.37	2	3.31	1.65	2.97	0.07
	Treatment * Timepoint	2	509.00	254.00	1.02	0.37	2	3.75	1.87	3.37	**0.05**
	Residuals	30	7502.00	250.00			29	16.12	0.56		
**GTR2A2**	Treatment	1	40.27	40.27	21.43	**6.62E-05**	1	1.09	1.09	2.54	0.12
	Timepoint	2	7.61	3.81	2.03	0.15	2	1.87	0.93	2.17	0.13
	Treatment * Timepoint	2	7.61	3.81	2.03	0.15	2	1.97	0.99	2.29	0.12
	Residuals	30	56.37	1.88			29	12.47	0.43		
**GTR3A1**	Treatment	1	0.00	0.00	0.04	0.84	1	0.48	0.48	1.66	0.21
	Timepoint	2	0.08	0.04	0.39	0.68	2	0.30	0.15	0.51	0.61
	Treatment * Timepoint	2	0.08	0.04	0.39	0.68	2	0.30	0.15	0.51	0.61
	Residuals	30	2.92	0.10			28	8.11	0.29		
**CYP79B2**	Treatment	1	5866.00	5866.00	61.96	**8.75E-09**	1	1.94	1.94	1.22	0.28
	Timepoint	2	415.00	208.00	2.19	0.13	2	1.32	0.66	0.41	0.67
	Treatment * Timepoint	2	415.00	208.00	2.19	0.13	2	1.46	0.73	0.46	0.64
	Residuals	30	2840.00	95.00			29	46.12	1.59		
**CYP83A1**	Treatment	1	6.10	6.10	12.18	**1.52E-03**	1	0.49	0.49	0.96	0.34
	Timepoint	2	1.91	0.96	1.91	0.17	2	0.25	0.13	0.24	0.79
	Treatment * Timepoint	2	1.91	0.96	1.91	0.17	2	0.25	0.13	0.25	0.78
	Residuals	30	15.03	0.50			29	14.96	0.52		

## Discussion

Our study revealed that local accumulation of total GSLs in response to *D. radicum* root feeding coincided with an increased local expression of both GSL biosynthesis and transporter genes in *B. rapa*. We observed similar patterns in both experiments, demonstrating the reproducibility of our results. We hypothesized that activation of GSL transport genes to transport GSLs from distal tissues would precede local *de novo* biosynthesis gene activity. However, we did not observe such temporal dynamics in our experiments. Similarly, we did not find that systemic GSL levels or transporter gene expression are suppressed in favor of locally increasing taproot levels. As predicted by the ODT, we found that GSL levels increased in the taproot in response to local root herbivory. For benzyl and indole GSL, this increase occurred one day after the start of herbivory, and lasted for the complete 7-day period of herbivory. Contrary to our hypothesis, we did not observe a decline in GSL levels in distal tissues during this period. This makes it unlikely that rapid re-allocation of distal GSLs from the shoots contributed to the increase of GSLs in the taproot.

Over the two experiments, the increase of total GSL levels in the taproot was mainly driven by elevated indole GSL levels, whereas aliphatic GSL concentrations did not increase upon root herbivory in either experiment. The increase in indole GSL levels in response to feeding by the specialist *D. radicum* as observed in this study is comparable to that of *B. rapa* to herbivory by the generalist *Anomala cuprea* (Tsunoda et al., 2018). This suggests that chewing root-herbivores with different degrees of host-plant specialization induce similar GSL profiles in *B. rapa*. In addition to indole GSL accumulation, the levels of the benzyl GSL gluconasturtiin (2-phenylethylglucosinolate) also increased in response to root-herbivory. Gluconasturtiin is a dominant GSL present in roots of *Brassica* species (van Dam et al., 2009). Its breakdown product 2-phenylethyl ITC, which is formed upon root fly feeding (Crespo et al., 2012), has chemical traits that are advantageous in soil conditions, such as low volatility and hydrophobicity (Sarwar et al., 1998; Laegdsmand et al., 2007). In addition, gluconasturtiin can have a negative effect on belowground herbivore performance, as was shown for *D. radicum* larvae feeding on *Barberea vulgaris* plants with differential GSL profiles (Van Leur et al., 2008). Pupae of *D. radicum* larvae feeding on *B. vulgaris* roots with gluconasturtiin as the dominant GSL where underdeveloped compared to those feeding from plants that mainly produced glucobarbarin (2(S)-OH-2-phenylethylglucosinolate). Although aliphatic GSLs have shown to play an important role in immunity against several chewing insect species in shoot tissues (Beekwilder et al., 2008; Müller et al., 2010; Jeschke et al., 2017), we did not find an induction of aliphatic GSL accumulation in response to the root-herbivore *D. radicum*. These observations are in accordance with the theory that shoot and root tissues rely on distinct GSL profiles as chemical defenses against a different community of chewing insect herbivores (Tsunoda and van Dam, 2017).

The accumulation patterns that we observed during root-herbivory can be largely explained by local expression levels of biosynthesis genes. The accumulation of indole GSLs in the taproot was preceded by an increased local expression of the biosynthesis gene CYP79B2, which lasted for the entire three-day period of herbivory. Interestingly, we also observed an initial increase in CYP83A1 expression after 12 h, suggesting that root-herbivory also would increase biosynthesis of aliphatic GSLs. However, CYP83A1 transcript levels dropped back to control conditions one day after the start of herbivory. This decrease in CYP83A1 expression coincided with a rise in CYP79B2 expression, suggesting that crosstalk occurred between the indole- and aliphatic GSL biosynthesis pathways. Such crosstalk between GSL biosynthetic pathways in favor of indole GSL synthesis was also observed in interactions between *A. thaliana* and the oomycete pathogen *Phytophthora brassicae* (Schlaeppi et al., 2010). Because specialist herbivores, such as *D. radicum*, may be able to detoxify GSL-based defenses, crosstalk might serve to switch from production of aliphatic GSLs which are ineffective against specialist herbivores, towards to the production of antimicrobial indole GSLs. Because of this antimicrobial effect of indole GSLs (Bednarek et al., 2009; Schlaeppi et al., 2010), we hypothesize that the observed accumulation of indole GSLs in the taproot may serve to prevent secondary infection by soil-borne pathogens. Root-herbivores generally cause damage to plant tissue over an extended period (Johnson et al., 2016), which increases secondary infections by microbial invaders. This may be particularly so for roots, as soils may contain up to 1 billion microbial cells per 10 grams of soil (Prosser, 2015). In *Arabidopsis thaliana*, indole GSLs work in concert with the structurally related phytoalexin camalexin to battle pathogen infection (Schlaeppi et al., 2010). In this case, indole GSLs slow down the infection cycle of pathogens by limiting penetration of the epidermal cell layer, after which camalexin serves as a late-acting antimicrobial defense-barrier. Although camalexin is not present in *B. rapa*, there are other phytoalexins that the species produces in response to biotic stressors. A prominent phytoalexin in several *Brassica* species is brassinin (Klein and Sattely, 2017), which is synthesized from the unstable ITCs that are formed during hydrolysis of glucobrassicin (indol-3-ylmethylglucosinolate) (Pedras et al., 2009; Bednarek, 2012). Glucobrassicin is one of the indole GSL that was induced by *D. radicum* feeding in this study. In addition, the biosynthesis of indole GSLs is closely linked to that of indole-3-acetic acid (IAA) (Malka and Cheng, 2017), the most commonly occurring plant hormone belonging to the auxin class (reviewed in Zhao, 2010). Next to its role in growth and development, IAA is a regulator of callus formation in response to wounding. By forming a physical barrier, callus can reduce infection by closing the wounds, thereby reducing infection by pathogens (Ikeuchi et al., 2013). Last but not least, the root flies themselves bring along microbial communities in their guts, which may help them to overcome their host plant’s chemical defenses (Welte et al., 2016). Some of these gut microbes, for example *Pectobacterium* spp, are also root pathogens in *Brassica* crops (van den Bosch et al., 2018). Taken together, it is very likely that the responses triggered by root herbivory are partly triggered by and targeted to microbial pathogens (Sellam et al., 2007). Brassinin and IAA are therefore interesting targets for future studies on interactions with root-herbivores and related microbial infections in *Brassica*.

Next to the induction of biosynthesis, herbivory induced the local expression of transporters GTR1 and GTR2, whereas expression of GTR3 was not affected. Since the induction of GTRs preceded the rise in indole GSL concentration, this suggests that active transport from distal tissues potentially plays a role in local accumulation of indole GSLs. However, contrary to our hypotheses, we did not observe any changes in GSL concentrations or expression of GTRs in distal tissues. This implies that local biosynthesis, and not transport from distal tissues, drives the accumulation of indole GSLs in response to root-herbivory. To confirm this hypothesis, the origin of GSLs that accumulated in the taproot should be studied by excluding the effects of transport from distal organs. This could be achieved by introducing isotope labelled GSLs or precursors into organs distal from the taproot, after which their distribution upon root herbivory can be studied. Next to transporting GSLs towards distal plant parts, GTRs also play a role in the retention of GSLs in designated plant parts (Jørgensen et al., 2017). An alternative hypothesis is that the increased expression of GTRs we observed in the taproot serves to prevent allocation of indole GSLs to plant parts that are not under imminent threat. By using specific GTR-knockout mutants, the role of transporter proteins in the retention of GSLs in the taproot can be studied. In conclusion, our study suggests that both biosynthesis and transport processes play a role in the accumulation of GSLs in the taproot during root-herbivory. However, the exact function and relative importance of transporters upon belowground plant–herbivore interactions needs to be confirmed in future studies.

## Data Availability Statement

All datasets generated for this study are included in the article/**Supplementary Material**.

## Author Contributions

TT, ND, and AT designed the experiments. TT performed the first experiment. AT and TT performed molecular analysis of the first experiment. AT and AVM carried out chemical analysis of the first experiment. AT and AVM performed the second experiment as well as molecular and chemical analyses. AT performed data analysis, supervised by TT and ND. AT, ND, and TT interpreted the results. AT wrote the manuscript under supervision of ND and TT. AM and RS assisted in molecular and chemical analyses and together with AVM provided helpful feedback on the manuscript.

## Funding

The authors acknowledge support from the Open Access Fund of the ThULB (Thüringer Universitäts-und Landesbibliothek Jena) and the iDiv Open Science Publication Fund for their contribution to the publication fee.

## Conflict of Interest

The authors declare that the research was conducted in the absence of any commercial or financial relationships that could be construed as a potential conflict of interest.

## References

[B1] Andersen T. G. Nour-Eldin H. H. Fuller V. L. Olsen C. E. Burow M. Halkier B. A. (2013). Integration of biosynthesis and long-distance transport establish organ-specific glucosinolate profiles in vegetative *Arabidopsis* . Plant Cell 25, 3133–3145. 10.1105/tpc.113.110890 23995084 PMC3784604

[B2] Bednarek P. Piślewska-Bednarek M. Svatoš A. Schneider B. Doubský J. Mansurova M. (2009). A glucosinolate metabolism pathway in living plant cells mediates broad-spectrum antifungal defense. Science (80-.) 323, 101 LP–106. 10.1126/science.1163732 19095900

[B3] Bednarek P. (2012). Sulfur-containing secondary metabolites from *Arabidopsis thaliana* and other Brassicaceae with function in plant immunity. ChemBioChem 13, 1846–1859. 10.1002/cbic.201200086 22807086

[B4] Beekwilder J. van Leeuwen W. van Dam N. M. Bertossi M. Grandi V. Mizzi L. (2008). The impact of the absence of aliphatic glucosinolates on insect herbivory in *Arabidopsis* . PloS One 3, e2068. 10.1371/journal.pone.0002068 18446225 PMC2323576

[B5] Bejai S. Fridborg I. Ekbom B. (2012). Varied response of *Spodoptera littoralis* against *Arabidopsis thaliana* with metabolically engineered glucosinolate profiles. Plant Physiol. Biochem. 50, 72–78. 10.1016/j.plaphy.2011.07.014 21835629

[B6] Burow M. Halkier B. A. (2017). How does a plant orchestrate defense in time and space? Using glucosinolates in *Arabidopsis* as case study. Curr. Opin. Plant Biol. 38, 142–147. 10.1016/j.pbi.2017.04.009 28575680

[B7] Crespo E. Hordijk C. A. de Graaf R. M. Samudrala D. Cristescu S. M. Harren F. J. M. (2012). On-line detection of root-induced volatiles in *Brassica nigra* plants infested with *Delia radicum L.* root fly larvae. Phytochemistry 84, 68–77. 10.1016/j.phytochem.2012.08.013 22995928

[B8] Danner H. Brown P. Cator E. A. Harren F. J. M. van Dam N. M. Cristescu S. M. (2015). Aboveground and belowground herbivores synergistically induce volatile organic sulfur compound emissions from shoots but not from roots. J. Chem. Ecol. 41, 631–640. 10.1007/s10886-015-0601-y 26195194 PMC4525197

[B9] Feller C. Bleiholder H. Buhr L. Hack H. Hess M. Klose R. (1995). Phänologische Entwicklungsstadien von Gemüsepflanzen II. Fruchtgemüse und Hülsenfrüchte Codierung und Beschreibung nach der erweiterten BBCH-Skala - mit Abbildungen.

[B10] Grosser K. van Dam N. M. (2017). A straightforward method for glucosinolate extraction and analysis with high-pressure liquid chromatography (HPLC). J. Vis. Exp., (121) e55425. 10.3791/55425 PMC5409297 28362416

[B11] Ikeuchi M. Sugimoto K. Iwase A. (2013). Plant callus: mechanisms of induction and repression. Plant Cell 25, 3159–3173. 10.1105/tpc.113.116053 24076977 PMC3809525

[B12] Jørgensen M. E. Nour-Eldin H. H. Halkier B. A. (2015). Transport of defense compounds from source to sink: lessons learned from glucosinolates. Trends Plant Sci. 20, 508–514. 10.1016/j.tplants.2015.04.006 25979806

[B13] Jørgensen M. E. Xu D. Crocoll C. Ramírez D. Motawia M. S. Olsen C. E. (2017). Origin and evolution of transporter substrate specificity within the NPF family. Elife 6, e19466. 10.7554/eLife.19466 28257001 PMC5336358

[B14] Jeschke V. Kearney E. E. Schramm K. Kunert G. Shekhov A. Gershenzon J. (2017). How glucosinolates affect generalist lepidopteran larvae: growth, development and glucosinolate metabolism. Front. Plant Sci. 8, 1995. 10.3389/fpls.2017.01995 29209354 PMC5702293

[B15] Johnson S. N. Erb M. Hartley S. E. (2016). Roots under attack: contrasting plant responses to below- and aboveground insect herbivory. New Phytol. 210, 413–418. 10.1111/nph.13807 26781566

[B16] Kim J. H. Jander G. (2007). *Myzus persicae* (green peach aphid) feeding on *Arabidopsis* induces the formation of a deterrent indole glucosinolate. Plant J. 49, 1008–1019. 10.1111/j.1365-313X.2006.03019.x 17257166

[B17] Kim J. H. Lee B. W. Schroeder F. C. Jander G. (2008). Identification of indole glucosinolate breakdown products with antifeedant effects on *Myzus persicae* (green peach aphid). Plant J. 54, 1015–1026. 10.1111/j.1365-313X.2008.03476.x 18346197

[B18] Kissen R. Rossiter J. T. Bones A. M. (2009). The `mustard oil bomb’: not so easy to assemble?! Localization, expression and distribution of the components of the myrosinase enzyme system. Phytochem. Rev. 8, 69–86. 10.1007/s11101-008-9109-1

[B19] Klein A. P. Sattely E. S. (2017). Biosynthesis of cabbage phytoalexins from indole glucosinolate. Proc. Natl. Acad. Sci. U. S. A. 114, 1910–1915. 10.1073/pnas.1615625114 28154137 PMC5338394

[B20] Laegdsmand M. Gimsing A. L. Strobel B. W. Sørensen J. C. Jacobsen O. H. Hansen H. C. B. (2007). Leaching of isothiocyanates through intact soil following simulated biofumigation. Plant Soil 291, 81–92. 10.1007/s11104-006-9176-2

[B21] Livak K. J. Schmittgen T. D. (2001). Analysis of relative gene expression data using real-time quantitative PCR and the 2–ΔΔCT method. Methods 25, 402–408. 10.1006/meth.20011262 11846609

[B22] Madsen S. R. Olsen C. E. Nour-Eldin H. H. Halkier B. A. (2014). Elucidating the role of transport processes in leaf glucosinolate distribution. Plant Physiol. 166, 1450–1462. 10.1104/pp.114.246249 25209984 PMC4226354

[B23] Malka S. K. Cheng Y. (2017). Possible interactions between the biosynthetic pathways of indole glucosinolate and auxin. Front. Plant Sci. 8, 2131. 10.3389/fpls.2017.02131 29312389 PMC5735125

[B24] McKey D. (1974). Adaptive patterns in alkaloid physiology. Am. Nat. 108, 305–320. 10.1086/282909

[B25] Meldau S. Erb M. Baldwin I. T. (2012). Defence on demand: mechanisms behind optimal defence patterns. Ann. Bot. 110, 1503–1514. 10.1093/aob/mcs212 23022676 PMC3503495

[B26] Müller R. de Vos M. Sun J. Y. (2010). Differential effects of indole and aliphatic glucosinolates on lepidopteran herbivores. J. Chem. Ecol. 36, 905–913. 10.1007/s10886-010-9825-z 20617455

[B27] Nour-Eldin H. H. Andersen T. G. Burow M. Madsen S. R. Jorgensen M. E. Olsen C. E. (2012). NRT/PTR transporters are essential for translocation of glucosinolate defence compounds to seeds. Nature 488, 531–534. 10.1038/nature11285 22864417

[B28] Oñate-Sánchez L. Vicente-Carbajosa J. (2008). DNA-free RNA isolation protocols for *Arabidopsis thaliana*, including seeds and siliques. BMC Res. Notes 1, 93. 10.1186/1756-0500-1-93 18937828 PMC2613888

[B29] Ohnmeiss T. E. Baldwin I. T. (2000). Optimal defense theory predicts the ontogeny of an induced nicotine defense. Ecology 81, 1765–1783. 10.1890/0012-9658(2000)081[1765:ODTPTO]2.0.CO;2

[B30] Pedras M. S. C. Okinyo-Owiti D. P. Thoms K. Adio A. M. (2009). The biosynthetic pathway of crucifer phytoalexins and phytoanticipins: de novo incorporation of deuterated tryptophans and quasi-natural compounds. Phytochemistry 70, 1129–1138. 10.1016/j.phytochem.2009.05.015 19560792

[B31] Piasecka A. Jedrzejczak-Rey N. Bednarek P. (2015). Secondary metabolites in plant innate immunity: conserved function of divergent chemicals. New Phytol. 206, 948–964. 10.1111/nph.13325 25659829

[B32] Prosser J. I. (2015). Dispersing misconceptions and identifying opportunities for the use of *omics* in soil microbial ecology. Nat. Rev. Microbiol. 13, 439. 10.1038/nrmicro3468 26052662

[B33] RStudio Team. (2018). RStudio: Integrated Development for R. (Boston, MA: RStudio, Inc.). Available at: http://www.rstudio.com/.

[B34] Sønderby I. E. Geu-Flores F. Halkier B. A. (2010). Biosynthesis of glucosinolates - gene discovery and beyond. Trends Plant Sci. 15, 283–290. 10.1016/j.tplants.2010.02.005 20303821

[B35] Sarwar M. Kirkegaard J. A. Wong P. T. W. Desmarchelier J. M. (1998). Biofumigation potential of brassicas. Plant Soil 201, 103–112. 10.1023/A:1004381129991

[B36] Schlaeppi K. Bodenhausen N. Buchala A. Mauch F. Reymond P. (2008). The glutathione-deficient mutant pad2-1 accumulates lower amounts of glucosinolates and is more susceptible to the insect herbivore *Spodoptera littoralis* . Plant J. 55, 774–786. 10.1111/j.1365-313X.2008.03545.x 18466300

[B37] Schlaeppi K. Abou-Mansour E. Buchala A. Mauch F. (2010). Disease resistance of *Arabidopsis* to *Phytophthora brassicae* is established by the sequential action of indole glucosinolates and camalexin. Plant J. 62, 840–851. 10.1111/j.1365-313X.2010.04197.x 20230487

[B38] Schoonhoven L. M. van Loon J. A. Dicke M. (2005). Insect-Plant Biology (Oxford, UK: Oxford University Press).

[B39] Sellam A. Iacomi-Vasilescu B. Hudhomme P. Simoneau P. (2007). In vitro antifungal activity of brassinin, camalexin and two isothiocyanates against the crucifer pathogens *Alternaria brassicicola* and *Alternaria brassicae* . Plant Pathol. 56, 296–301. 10.1111/j.1365-3059.2006.01497.x

[B40] Textor S. Gershenzon J. (2009). Herbivore induction of the glucosinolate–myrosinase defense system: major trends, biochemical bases and ecological significance. Phytochem. Rev. 8, 149–170. 10.1007/s11101-008-9117-1

[B41] Tsunoda T. van Dam N. M. (2017). Root chemical traits and their roles in belowground biotic interactions. Pedobiologia 65, 58–67. 10.1016/j.pedobi.2017.05.007

[B42] Tsunoda T. Krosse S. van Dam N. M. (2017). Root and shoot glucosinolate allocation patterns follow optimal defence allocation theory. J. Ecol. 105, 1256–1266. 10.1111/1365-2745.12793

[B43] Tsunoda T. Grosser K. van Dam N. M. (2018). Locally and systemically induced glucosinolates follow optimal defence allocation theory upon root herbivory. Funct. Ecol. 32, 2127–2137. 10.1111/1365-2435.13147

[B44] Tytgat T. O. G. Verhoeven K. J. F. Jansen J. J. Raaijmakers C. E. Bakx-Schotman T. McIntyre L. M. (2013). Plants know where it hurts: root and shoot jasmonic acid induction elicit differential responses in *Brassica oleracea* . PloS One 8, e65502–e65502. 10.1371/journal.pone.0065502 23776489 PMC3679124

[B45] van Dam N. M. Raaijmakers C. E. (2006). Local and systemic induced responses to cabbage root fly larvae (*Delia radicum*) in *Brassica nigra* and *B. oleracea* . Chemoecology 16, 17–24. 10.1007/s00049-005-0323-7

[B46] Van Dam N. M. Witjes L. Svatoš A. (2004). Interactions between aboveground and belowground induction of glucosinolates in two wild *Brassica* species. New Phytol. 161, 801–810. 10.1111/j.1469-8137.2004.00984.x 33873723

[B47] van Dam N. M. Tytgat T. O. G. Kirkegaard J. A. (2009). Root and shoot glucosinolates: a comparison of their diversity, function and interactions in natural and managed ecosystems. Phytochem. Rev. 8, 171–186. 10.1007/s11101-008-9101-9

[B48] van den Bosch T. J. M. Tan K. Joachimiak A. Welte C. U. (2018). Functional profiling and crystal structures of isothiocyanate hydrolases found in gut-associated and plant-pathogenic bacteria. Appl. Environ. Microbiol. 84, e00478–e00418. 10.1128/AEM.00478-18 29752272 PMC6029094

[B49] Van Leur H. Raaijmakers C. E. Van Dam N. M. (2008). Reciprocal interactions between the cabbage root fly (*Delia radicum*) and two glucosinolate phenotypes of *Barbarea vulgaris* . Entomol. Exp. Appl. 128, 312–322. 10.1111/j.1570-7458.2008.00722.x

[B50] Welte C. U. de Graaf R. M. van den Bosch T. J. M. den Camp H. J. M. van Dam N. M. Jetten M. S. M. (2016). Plasmids from the gut microbiome of cabbage root fly larvae encode SaxA that catalyses the conversion of the plant toxin 2-phenylethyl isothiocyanate. Environ. Microbiol. 18, 1379–1390. 10.1111/1462-2920.12997 26234684

[B51] Wittstock U. Burow M. (2010). Glucosinolate breakdown in *Arabidopsis*: mechanism, regulation and biological significance. Arab. B. 8, e0134–e0134. 10.1199/tab0134 PMC3244901 22303260

[B52] Wittstock U. Gershenzon J. (2002). Constitutive plant toxins and their role in defense against herbivores and pathogens. Curr. Opin. Plant Biol. 5, 300–307. 10.1016/S1369-5266(02)00264-9 12179963

[B53] Zhao Y. (2010). Auxin biosynthesis and its role in plant development. Annu. Rev. Plant Biol. 61, 49–64. 10.1146/annurev-arplant-042809-112308 20192736 PMC3070418

